# Antibodies to Polymorphic Invasion-Inhibitory and Non-Inhibitory Epitopes of *Plasmodium falciparum* Apical Membrane Antigen 1 in Human Malaria

**DOI:** 10.1371/journal.pone.0068304

**Published:** 2013-07-05

**Authors:** Cleopatra K. Mugyenyi, Salenna R. Elliott, Fiona J. McCallum, Robin F. Anders, Kevin Marsh, James G. Beeson

**Affiliations:** 1 Kenya Medical Research Institute, Centre for Geographic Medicine, Coast, Kilifi, Kenya; 2 Burnet Institute, Melbourne, Victoria, Australia; 3 The Walter and Eliza Hall Institute of Medical Research, Victoria, Australia; 4 LaTrobe University, Victoria, Australia; 5 Monash University, Victoria, Australia; London School of Hygiene and Tropical Medicine, United Kingdom

## Abstract

**Background:**

Antibodies to *P. falciparum* apical membrane protein 1 (AMA1) may contribute to protective immunity against clinical malaria by inhibiting blood stage growth of *P. falciparum*, and AMA1 is a leading malaria vaccine candidate. Currently, there is limited knowledge of the acquisition of strain-specific and cross-reactive antibodies to AMA1 in humans, or the acquisition of invasion-inhibitory antibodies to AMA1.

**Methodology/Findings:**

We examined the acquisition of human antibodies to specific polymorphic invasion-inhibitory and non-inhibitory AMA1 epitopes, defined by the monoclonal antibodies 1F9 and 2C5, respectively. Naturally acquired antibodies were measured in cohorts of Kenyan children and adults. Antibodies to the invasion-inhibitory 1F9 epitope and non-inhibitory 2C5 epitope were measured indirectly by competition ELISA. Antibodies to the 1F9 and 2C5 epitopes were acquired by children and correlated with exposure, and higher antibody levels and prevalence were observed with increasing age and with active *P. falciparum* infection. Of note, the prevalence of antibodies to the inhibitory 1F9 epitope was lower than antibodies to AMA1 or the 2C5 epitope. Antibodies to AMA1 ectodomain, the 1F9 or 2C5 epitopes, or a combination of responses, showed some association with protection from *P. falciparum* malaria in a prospective longitudinal study. Furthermore, antibodies to the invasion-inhibitory 1F9 epitope were positively correlated with parasite growth-inhibitory activity of serum antibodies.

**Conclusions/Significance:**

Individuals acquire antibodies to functional, polymorphic epitopes of AMA1 that may contribute to protective immunity, and these findings have implications for AMA1 vaccine development. Measuring antibodies to the 1F9 epitope by competition ELISA may be a valuable approach to assessing human antibodies with invasion-inhibitory activity in studies of acquired immunity and vaccine trials of AMA1.

## Introduction

Immunity to *Plasmodium falciparum* malaria eventually develops after repeated exposure to infection, and is characterised by control of blood-stage parasitemia and prevention of clinical illness and severe complications [1]. Antibodies play a major role in acquired immunity, however the key targets and mechanisms of action of protective human antibodies are not well understood [2]. *P. falciparum* merozoites invade erythrocytes during blood stage replication, and antibodies that inhibit invasion by targeting merozoite antigens are believed to be important for acquired immunity [3], [4]. Identifying targets of protective antibodies in humans and understanding the mechanisms by which antibodies to merozoite antigens protect against malaria is essential for the development of blood-stage malaria vaccines, as well as for developing approaches to monitor immunity in populations, assess the impact of malaria control interventions on immunity, and identify populations at high risk of malaria.

The merozoite antigen apical membrane antigen 1 (AMA1) is a leading vaccine candidate and appears to be an important target of acquired immunity. It plays a key role in erythrocyte invasion [5]–[8], and antibodies raised against AMA1 or affinity-purified AMA1 antibodies from naturally exposed individuals inhibit merozoite invasion in vitro [9]–[15]. Immunization of animals with AMA1 can protect against blood stage challenge with the homologous strain, but less effectively against heterologous strains due to antigenic diversity (reviewed [15]). Antibodies to AMA1 are typically highly prevalent amongst people in malaria endemic populations [16]–[21]. Some longitudinal studies have associated antibodies to recombinant AMA1 measured by ELISA with reduced risk of malaria [17], [21]–[23]; however others have found little or no protective effect [24]–[26]. A recent systematic review of longitudinal studies found a tendency towards a protective association amongst studies that met rigorous quality criteria for inclusion [4]. In a recent clinical trial of the vaccine FMP2.1/AS02_A_ containing recombinant AMA1 of the 3D7 strain, there was no significant protection against clinical malaria overall, but there was a significant reduction in risk of clinical malaria caused by parasites expressing vaccine-like AMA1 alleles, suggesting strain-specific protective efficacy [27], [28]. These results support the development of AMA1 as a malaria vaccine, but highlight the need to better understand antigenic diversity of AMA1 and the functional activity of antibodies against AMA1.

The crystal structure of AMA1 reveals a long hydrophobic trough in domain I that appears to be a binding site for proteins forming an erythrocyte invasion complex comprised of AMA1 and RON proteins [6], [7], [29], [30]. One end of this trough is flanked by many of the most polymorphic residues in the protein. These polymorphisms appear to have arisen due to diversifying selection and presumably allow the parasite to avoid invasion-inhibitory antibodies [31], [32]. Animal and in vitro studies indicate that immune responses targeting AMA1 are at least partly strain specific [9]–[12], [17], [19], [33]. Although there are a large number of different AMA1 alleles circulating in human populations, recent studies have suggested that the extent of antigenic diversity may be limited, as evidenced by substantial cross-inhibitory activity of antibodies to isolates expressing different AMA1 alleles [33], and sequence analyses suggesting that AMA1 alleles may be clustered into a small number of related groups [34]–[36].

Antibodies to AMA1 are thought to contribute to protective immunity by inhibiting erythrocyte invasion and blood-stage replication of *P. falciparum*. However, to date, it has not been possible to directly measure AMA1-specifc inhibitory antibodies among individuals in relation to protection from clinical malaria, and better understand the acquisition of inhibitory antibodies. Furthermore, knowledge on the acquisition of antibodies to polymorphic and conserved epitopes in relation to immunity is limited [17], [22], [23]. To address these questions, we tested the ability of human antibodies to inhibit the binding of two monoclonal antibodies (mAbs) that target polymorphic epitopes of AMA1, the invasion-inhibitory 1F9 epitope and the non-inhibitory 2C5 epitope. These mAbs were generated to the 3D7 strain of *P. falciparum* AMA1 by standard methods [37], [38]. 1F9 binds to a conformation-dependent polymorphic epitope in domain I of AMA1 in a strain specific manner [37], [38]. The binding site includes part of the hydrophobic trough and some of the adjacent loops on the AMA1 surface [39]. 1F9 inhibits *in vitro* growth of 3D7 and D10 parasite strains, but not HB3 or W2mef [38], and has been shown to inhibit binding of AMA1 by the rhoptry neck protein, RON2, a key interaction that is required for formation of the tight junction during invasion [7], [8]. Mutations of polymorphic residues in the cluster 1 loop of domain I (C1-L) of AMA1 abrogate binding of 1F9 [39]. An in vitro study suggested that polymorphic residues within the Cl-L region contribute to antigen escape [40], and amino acid changes in this region between consecutive infecting strains have been linked to development of clinical symptoms [36]. This region of AMA1 also appeared to be targeted by human vaccine-induced antibodies in the recent phase 2 clinical trial [27], [28]. Thus polymorphic residues within the C1-L region that are recognised by the 1F9 mAb appear to be important immune targets. The 2C5 mAb recognises a complex conformational epitope that has not been fully characterised [38]. 2C5 shows the same pattern of strain-specific reactivity as 1F9, but does not inhibit invasion, thus the 2C5 epitope must be distinct from that of 1F9 [38].

Using the 1F9 and 2C5 mAb in competition ELISAs we were able to indirectly measure naturally acquired antibodies to two polymorphic epitopes of AMA1, one of which is recognised as a functional epitope and a potentially important immune target. Our aims were to define the acquisition of antibodies to these epitopes in relation to age, parasitemia, and markers of exposure to malaria, examine the association between antibodies and risk of malaria in a longitudinal cohort of children, and assess the relationship between antibodies to the inhibitory 1F9 epitope and growth inhibitory antibodies.

## Materials and Methods

### Ethics Statement

Ethical clearance was provided by the Ethics Committee of the Kenya Medical Research Institute, the Human Research Ethics Committee of the Walter and Eliza Hall Institute and the Alfred Hospital Human Research and Ethics Committee (for the Burnet Institute). Written informed consent was provided by all subjects or their guardians.

### Study Populations, Sample Collection and Surveillance

Samples were obtained from two villages, Chonyi and Ngerenya, located in Kilifi district, Kenya [17], [41]. The two study sites were included to allow an evaluation of antibodies in populations exposed to different levels of transmission intensity. The Chonyi cohort was included to enable the associations between antibodies and malaria incidence and risk to be examined, and the Ngerenya cohort was included to enable an evaluation of the relationship between antibodies to specific epitopes and parasite growth-inhibitory activity of serum antibodies because of the availability of sufficient serum volumes. Insufficient sample volumes were available to test growth inhibitory activity using Chonyi samples. Biannual malaria transmission occurs during the rainy seasons of May-July and November-December. At the time of sample collection, Chonyi had medium-high transmission with an entomologic inoculation rate (EIR) of 20–100 infective bites per year [23], [42], [43] and Ngerenya was a low-medium transmission area with an EIR of 10 infective bites per year.

In the Chonyi cohort, participants were enrolled and finger-prick blood was collected in October 2000 for baseline antibody measurements and blood smears for microscopy. Asymptomatic parasitemia was not treated. Participants were followed by active and passive case detection for symptomatic illness for 210 days. Blood-smears for malaria diagnosis were performed on any symptomatic individuals. Malaria was defined as a temperature greater than 37.5°C with a parasitemia above 2500 parasites/µl. The cohort has been described in detail elsewhere [17], [23], [41], [44]. We included 248 children aged 6 months to 10 years (<1 yr, n = 20; 1–2 yrs n = 51; 3–4 yr, n = 52; 5–6 yrs n = 52; 7–8 yrs n = 53; 9–10 yrs n = 20) and a selection of older children (11–15 years, n = 10) and adults (aged 16–53; n = 17) for comparison of antibody responses to children. Children aged 1–10 years were the primary focus of our studies because this was the period during which immunity was acquired.

Samples were collected from subjects in the Ngerenya study site in September 1998, including 150 children and adults (2–5 years, n = 43; 6–14 years, n = 57; 18–81 years, n = 50) [45]. These sera were used to examine associations between antibody responses to AMA1 and the 1F9 epitope and growth inhibitory activity by serum antibodies. Sera from 20 non-exposed British and Australian donors were used as reference controls.

### Monoclonal Antibodies

Monoclonal antibodies (mAb) to the AMA1 epitopes 1F9 and 2C5 were used [38]. The mAb were generated previously by immunization of mice with recombinant refolded 3D7 AMA1 and were purified by protein G chromatography [37], [39]. Reactivity of the mAb to AMA1 was established previously by ELISA and immunofluorescence on 3D7 schizonts and merozoites [37].

### Standard ELISA

Measurement of serum antibodies to the 3D7 allele of AMA1 and schizont extract was carried out using standard enzyme linked immunosorbent assay (ELISA) [21], [46]. AMA1 was expressed as a His-tagged recombinant protein in *Escherichia coli* using the full ectodomain of 3D7 (21). Schizont protein extract was prepared from *P. falciparum* (A4) by sonicating highly synchronous parasites on ice [47]. Ninety six well ELISA plates were coated with 0.5 µg/ml of AMA1 or a 1∶1000 dilution of schizont protein extract overnight at 4°C. Plates were washed with PBS, 0.05% Tween 20 and blocked with PBS/Tween 20/10% skim milk for 2 hours. Serum diluted in sample buffer (PBS/Tween 20/5% skim milk) at 1∶2000 dilution (AMA1) or 1∶1000 (schizont extract) was added to duplicate wells and incubated at room temperature for one hour. Bound IgG was detected using secondary antibody (HRP conjugated polyclonal anti-human IgG, Dako) diluted in sample buffer at 1∶2500 (AMA1) or 1∶2000 (schizont extract) and incubated for one hour at room temperature, then visualised by incubating with ABTS [2,2-azinobis (3-ethylbenzthiazolinesulfonic acid)] substrate system (Sigma-Aldrich, Australia) in the dark for 15 minutes. Measurement of IgM antibodies to recombinant AMA1 was carried out using the same technique with a 1∶100 serum dilution. IgM antibodies were detected using HRP conjugated anti-human IgM (Chemicon, AP114P) at a 1∶2500 dilution. The cut-off selected for antibody positivity was the mean +3 standard deviations of negative control sera from non-exposed donors tested in parallel, consistent with previous approaches [21], [48].

### Competition ELISA

Epitope-specific antibodies to AMA1 (3D7 allele) were measured indirectly using competition ELISAs, which measured the ability of serum antibodies to compete with binding of epitope-specific monoclonal antibodies, 1F9 and 2C5. ELISA plates were coated with 0.5 µg/ml of AMA1 overnight at 4°C. Wells were washed three times and incubated with blocking buffer at 37°C for two hours. The optimal concentrations of mAb 1F9 and 2C5 were established in preliminary experiments by performing serial dilutions of each mAb. Standard curves were plotted and the optimum amount of each mAb was selected as 0.2 µg/ml. This value was close to saturation but still on the linear part of the curve such that there was enough mAb to occupy most specific sites in the absence of competing antibody. Sera were diluted in sample buffer together with the appropriate mAb (serum at 1∶250 with 1F9 and 1∶500 with 2C5; pilot experiments were performed to determine the optimal serum dilution for competition ELISA with each mAb). The serum/mAb mix was added to duplicate wells and incubated at 37°C for one hour. The plates were washed three times and incubated with secondary antibody (HRP conjugated rabbit mouse IgG diluted at 1∶2500 in sample buffer) for one hour at room temperature. After three washes, plates were incubated in the dark with ABTS substrate system (Sigma-Aldrich, Australia) for 15 minutes. The development reaction was stopped using 1% SDS. Optical density was measured at 405 nm. Results were expressed as the percentage inhibition relative to binding of the monoclonal antibody in the absence of serum ((OD no serum – OD serum)/OD no serum x100), where high % inhibition values indicated high levels of epitope-specific antibodies in the serum sample. The cut-off used to define inhibitory samples was the mean +2 standard deviations of negative control sera tested in parallel.

### Growth Inhibition Assay

*In vitro* inhibition of *P. falciparum* growth by human serum was tested by growth inhibition assay (GIA) with the growth of parasites expressed as a percentage of the maximal growth achieved in control wells. Assays were performed using established methods [45], [49]. Heat inactivated dialysed sera were tested for inhibitory activity in 3077 sterile 96 well U-bottomed plates (Falcon) on highly synchronous trophozoite stage *P. falciparum* 3D7 parasites. The cultures were diluted to a starting parasitemia of 0.4–0.8% and 1% hematocrit at a volume of 25 µl per well with 2.5 µl of sample sera added into duplicate wells. Positive controls of pharmaceutical grade heparin (100 µg/ml) [50] and mAb 1F9 (10 µg/ml) [38] were included on each assay plate with PBS alone as a negative control. The plate was gassed and incubated at 37°C in a humidified chamber. After 36–48 hours when the parasites were at the late ring or early trophozoite stage, the parasites were stained with 10 µg/ml ethidium bromide in the dark for 1 hour and parasitemia was measured by flow cytometry.

### Statistical Analysis

All analyses were performed using STATA version 9.2 (StataCorp, College Station, TX USA). Median antibody levels were compared using the Mann Whitney or Kruskal Wallis test. Chi square tests were used to assess associations between antibody prevalence and age or exposure. Correlations between antibody levels and parasite growth inhibition were determined using Spearman’s rank correlation.

Associations between antibodies and risk of symptomatic malaria (with parasitemia >2500/µl) were examined in children aged 1–10 years from the Chonyi cohort. Analysis was performed until day 210 of follow-up, with the first 14 days excluded to reduce the effect of baseline parasitemia accounting for malaria episodes in the follow-up period. Although some children had multiple episodes of malaria, only the first episode was included in the analysis. Survival analysis was performed among children from 1 year to 10 years of age (n = 228), because prior studies have shown that associations between antimalarial antibodies and protective immunity in this population are restricted to this age range, and adults were excluded to reduce the potential confounding effect of age and because few adults experienced malaria during follow-up (90% of clinical episodes occurred in children aged 10 years or less [23]). Children were classified into two groups, high or low responders, on the basis of being above or below the median level of activity in the cohort for each antibody parameter (IgG to AMA1, inhibition of 1F9 binding, or inhibition of 2C5 binding). Some samples gave negative values of inhibition (ie. enhanced binding of mAb) and these were not included in the survival analysis unless stated. Kaplan-Meier survival functions were used to investigate malaria rates. Differences in survival functions were tested using the Log-rank test. Differences in the likelihood of malaria at any point during follow up between high and low responders was analysed using Cox Proportional Hazards. The relationship between antibody levels and the risk of experiencing a clinical malaria episode in the 210 days post-sampling was analysed using univariate and multivariate generalised linear models (GLM). Possible confounding due to age was reduced by exclusion of adults from the study and by adjusting for age of children. Prior studies in the Chonyi cohort demonstrated that *P. falciparum* infection status at enrolment had a significant influence on the association between antibodies and risk of malaria [17]. Therefore, hazard ratios were also calculated for subjects stratified as parasite positive or negative at enrolment.

## Results

### Effects of Age, Parasitemic Status and Exposure on Acquisition of Epitope-specific Antibodies to AMA1

#### Chonyi cohort

We examined the acquisition of antibodies to the AMA1 ectodomain and the 1F9 and 2C5 epitopes in a cohort of 248 children (aged 6 months –10 years) in Chonyi and 27 older children and adults for comparison. The prevalence of AMA1 IgG was high at 82.3% (95% CI 77.6–87.1%). Antibodies to specific epitopes were less common, with 20.7% (95% CI 15.9–25.5) of children showing inhibition of 1F9 binding and 52.0% (95% CI 45.8–58.3%) inhibiting 2C5 binding by competition ELISA. Median anti-1F9 activity (0.4% (IQR −0.41–5.4%, range −14.6 to 65.1%) was lower than anti-2C5 activity (8.3%, (IQR 0.8–20.5%, range −18.0% to 93.6%). Some samples showed negative values of inhibition, which suggests enhanced binding of the monoclonal antibody to its epitope in the presence of serum (discussed further below). Inhibition of mAb binding to the 1F9 and 2C5 epitopes correlated with levels of antibodies to the AMA1 ectodomain (r = 0.57, P<0.001 and r = 0.79, P<0.001, respectively; Spearman’s rank correlation). There was also a positive correlation between anti-1F9 and anti-2C5 activity (r = 0.57, P<0.001, Spearman’s rank correlation).

**Figure 1:**
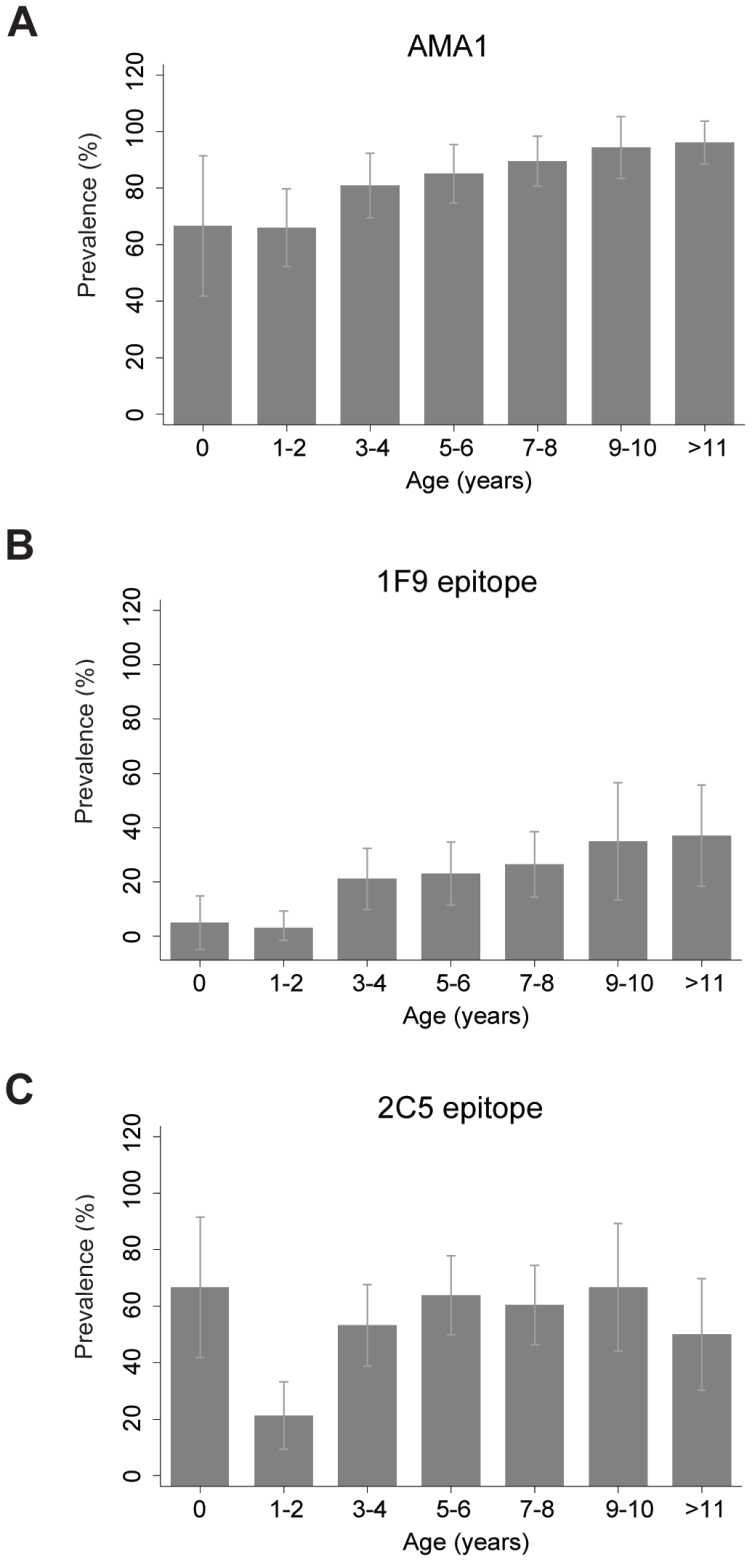
Prevalence of antibodies to AMA1 or specific epitopes by age group in the Chonyi cohort. Antibodies to recombinant AMA1 protein were tested by standard ELISA. Antibodies to specific AMA1 epitopes were measured by assessing the ability of human serum antibodies to inhibit the binding of monoclonal antibodies 1F9 and 2C5 in competition ELISA. (**A**) IgG to recombinant AMA1 (P<0.001), (**B**) inhibition of binding of mAb 1F9 epitope (P<0.001), (**C**) inhibition of binding of mAb 2C5 (P = 0.028). P values were calculated using the Chi square test for trend excluding age 0 (<1) years. Data represent the proportion of individuals with detectable IgG binding or inhibitory activity and error bars indicate 95% CI.

The prevalence of antibodies to the AMA1 ectodomain increased with increasing age (P<0.001, test for trend) and 96% individuals over 10 years were classified as positive for AMA1 IgG (Figure 1A). Antibodies to the 1F9 epitope were less prevalent than those to AMA1 or the 2C5 epitope in all age groups (Figure 1B). Only 40% of individuals above 10 years were classified as positive for 1F9 antibodies. There was a significant increase in prevalence of 1F9 antibodies with age (P<0.001, test for trend), and a weaker association between prevalence of 2C5 antibodies and increasing age (P = 0.028, test for trend) (Figure 1C). Maternal antibodies probably explain the high proportion of children under the age of 1 year with 2C5 antibodies (67%). Antibody levels were also significantly different among age groups (AMA1 IgG, P<0.001; anti-1F9 activity, P = 0.02; anti-2C5 activity, P<0.001, Kruskal Wallis). At sampling time, 43.2% of Chonyi children aged 1–10 years were parasitemic by light microscopy. There were significantly higher levels of antibodies to AMA1, and the 1F9 and 2C5 epitopes amongst parasitemic compared to aparasitemic individuals (P<0.01 for all antibodies, Mann Whitney U test). Reactivity to schizont protein extract was used as a measure of exposure to blood-stage *P. falciparum* [51]. There were significant positive correlations between reactivity to schizont extract and levels of antibodies to the AMA1 ectodomain (r = 0.54), the 1F9 epitope (r = 0.23) and the 2C5 epitope (r = 0.37) (Spearman’s rank correlation, P<0.001 for all associations).

#### Ngerenya cohort

Additional studies on antibodies to the AMA1 ectodomain and the 1F9 epitope were performed among 100 children (aged 2–14 years) and 50 adults of the Ngerenya cohort to allow comparison between antibodies to AMA1 and growth inhibitory antibodies (there was insufficient sample volume available to measure growth inhibitory antibodies in the Chonyi cohort) and to further evaluate antibody acquisition among children versus adults. The prevalence of IgG to the AMA1 ectodomain was lower than for Chonyi at 73.3% (95% CI 65.7–79.8%), despite inclusion of adults in this sample set, reflecting lower transmission intensity in this population. When the population was divided into three age groups (2–5 years n = 43; 6–14 years n = 57; 18–81 years n = 50), levels of AMA1 IgG were significantly different between groups, with lower levels amongst 2–5 year olds (median OD [IQR]: 2–5 yrs, 0.08 [0.04–0.55]; 6–14 yrs, 0.76 [0.16–1.21]; 18–81 yrs, 0.53 [0.14–0.85]; P<0.001, Kruskal Wallis). At the time of sampling, 39.3% of the population were parasitemic, and there was a significant difference in the level of IgG to AMA1 between aparasitemic and parasitemic individuals (median OD [IQR]: 0.26 [0.05–0.73] vs 0.82 [0.30–1.27], for aparasitemic and parasitemic groups, respectively; P<0.001 Mann Whitney test).

**Figure 2:**
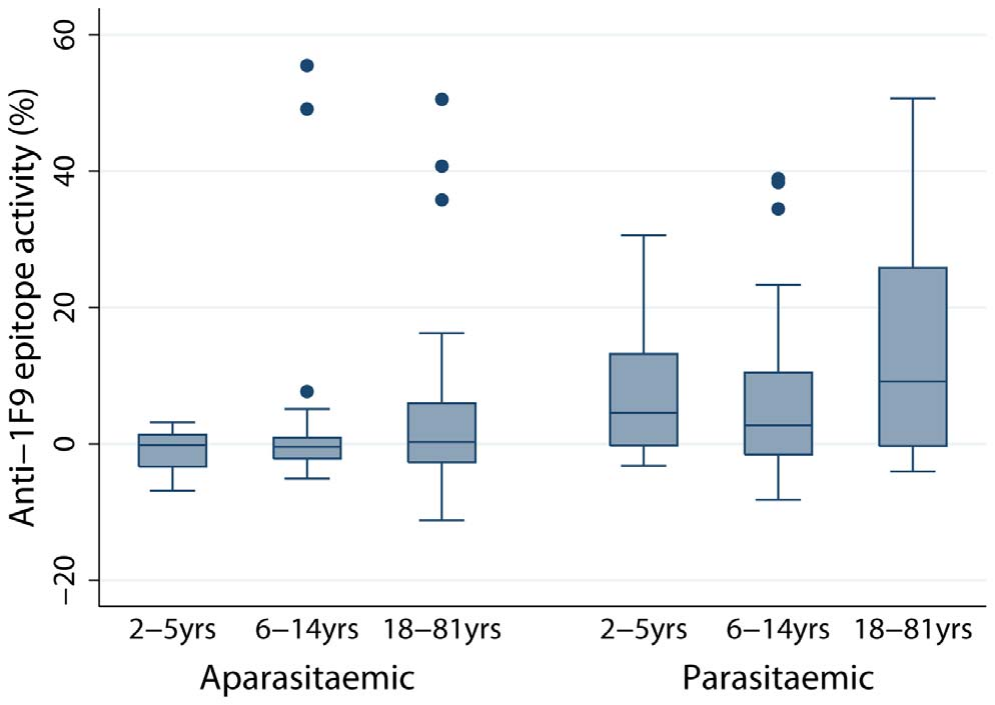
Levels of inhibition of 1F9-binding by human antibodies according to age group and infection status in the Ngerenya cohort. Values show the level of inhibition of 1F9 mAb binding to AMA1 by human serum antibodies, stratified by infection status at the time of sample collection and grouped according to age. Boxes indicate median and interquartile range, and whiskers represent 95% CI. Positive 1F9 inhibition was defined as inhibition of 1F9 binding>(mean +2 standard deviations of negative controls = 6.19%). Differences in the level of anti-1F9 activity among age groups was significant amongst aparasitemic individuals (P = 0.020, Kruskal Wallis test), but not amongst parasitemic individuals. The median level of anti-1F9 activity was lower amongst aparasitemic compared to parasitemic individuals (median inhibition [IQR] −0.1% [−2.8–1.7] vs 4.3% [−1.0–13.2], P = 0.002, Mann Whitney U test).

The level of inhibition of 1F9 binding (%) shown by most samples was low (median [IQR] = 0.5 [−2.2–5.2], range = −11.2 to 55.5), with negative values suggesting enhanced binding of 1F9 to its epitope. Thirty one (23.9%) individuals were defined as 1F9 inhibitors (inhibition of 1F9 binding>(mean +2 standard deviations of negative controls = 6.19%)). There was a moderate correlation between anti-1F9 activity and IgG to the AMA1 ectodomain (Spearman’s r = 0.48, P<0.001). When including all Ngerenya samples, neither levels of anti-1F9 activity or prevalence of 1F9 inhibitors was significantly different between the three age groups. However, after stratifying by parasitemic status, levels of anti-1F9 activity were significantly different between age groups amongst aparasitemic individuals (P = 0.020, Kruskal Wallis test), but not amongst parasitemic individuals (Figure 2). The median level of anti-1F9 activity was lower amongst aparasitemic compared to parasitemic individuals (Figure 2; median inhibition [IQR] −0.1% [−2.8–1.7] vs 4.3% [−1.0–13.2], P = 0.002, Mann Whitney U-test) and the prevalence of 1F9 inhibitors was also lower in aparasitemic individuals (15.0% versus 38.0% for parasitemic individuals; P = 0.003, Chi square test). The correlation between 1F9 antibody levels and schizont reactivity was modest (r = 0.26, P = 0.003) compared to that seen for AMA1 antibodies (r = 0.64, P<0.001).

### Association between Antibodies to AMA1 Epitopes and Protection from Clinical Malaria

The longitudinal follow-up of children in the Chonyi cohort allowed us to prospectively examine the association between antibodies at baseline and risk of clinical malaria during 6 months active follow-up. Two hundred and twenty eight children between the ages of 1 and 10 years were included in this analysis. Only children 1–10 years of age were included, since this is the group acquiring immunity in this community [41], and excluding adults helped reduce the potential confounding effect of age; infants were excluded because of the potential presence of maternally-transferred antibodies. Individuals were categorised into high and low responders according to whether their antibody response was above or below the median level for the group. As a previous study on the same cohort showed an association between protection from clinical malaria and antibodies to AMA1 was influenced by concurrent parasitemia at the time of sample collection [17], the effects of concurrent parasitemia and age were investigated.

Individuals with high responses to AMA1 or its specific epitopes had significantly lower rates of malaria when compared to those with low antibody responses (AMA1, P = 0.013; anti-1F9 activity, P = 0.006; anti-2C5 activity, P = 0.047; Log-rank test). The Cox proportional hazards model was used to calculate risk estimates and adjust for potential confounding. In the univariate analysis, younger age (P<0.001) and concurrent parasitemia at sampling (P = 0.04) were associated with significantly higher risks of clinical malaria. Associations between antibody levels and protection from malaria were assessed, with the dataset stratified by parasitemic status and the multivariate analysis adjusted for age. When antibody responses were analysed without taking into account parasitemic status, there was significantly less risk of a clinical malaria episode at any point during the 6 months after sampling in those with a high antibody response to AMA1 (HR = 0.36, P* = *0.02), the 1F9 epitope (HR = 0.21, P = 0.01), or the 2C5 epitope (HR = 0.38, P = 0.06) (Table 1). However, these associations were not statistically significant after adjusting for the age of the children. Amongst those who were parasite positive at sampling time, high responders to AMA1 (HR = 0.14, P = 0.01) and to the 2C5 epitope (HR = 0.08, P = 0.02) were much less likely to experience a clinical episode of malaria. They maintained this apparent protective association when the analysis was adjusted for age but the significance diminished (P = 0.08) (Table 1). Among the aparasitemic group, there was no significant association between antibodies to AMA1 or 2C5 and the risk of malaria. In contrast, antibodies to the 1F9 epitope showed a different pattern of association with protection. Antibodies to the 1F9 epitope were associated with a reduced risk of malaria in both the parasitemic and aparasitemic groups (HR = 0.29 and HR = 0.31, respectively) (Table 1), although these associations did not reach statistical significance in either group (P = 0.14), possibly because the smaller sample size leads to reduced statistical power; a statistically significant association between 1F9 antibodies and protection was only seen when all children were included in the analysis (HR = 0.21, P = 0.01).

**Table 1:** Association between antibody responses and risk of clinical malaria in the Chonyi cohort.

Antibody reactivity^1^		Cox proportional hazards		Adjusted Cox proportional hazards^2^	
		HR^3^	*P-*value	HR^3^	*P-*value
		**(95% CI)**		**(95% CI)**	
**All samples**	AMA1	0.36	0.02	0.81	0.66
		(0.15–0.84)		(0.31–2.10)	
	1F9 epitope	0.21	0.01	0.34	0.12
		(0.06–0.73)		(0.09–1.31)	
	2C5 epitope	0.38	0.06	0.85	0.77
		(0.14–1.04)		(0.28–2.55)	
**Parasitemic** 4	AMA1	0.14	0.01	0.23	0.08
		(0.03–0.64)		(0.05–1.18)	
	1F9 epitope	0.29	0.14	0.49	0.41
		(0.06–1.52)		(0.09–2.74)	
	2C5 epitope	0.08	0.02	0.15	0.08
		(0.01–0.66)		(0.02–1.27)	
**Aparasitemic** 4	AMA1	0.95	0.91	1.55	0.39
		(0.37–2.44)		(0.58–4.16)	
	1F9 epitope	0.31	0.14	0.38	0.23
		(0.06–1.49)		(0.08–1.85)	
	2C5 epitope	1.09	0.89	1.36	0.61
		(0.33–3.56)		(0.41–4.45)	

Notes.

1 Children were classified as high and low responders for antibodies based on being above or below the median level for the cohort.

2 Multivariable Cox proportional hazards adjusted for age.

3 Hazard ratios show the risk of *P. falciparum* malaria over a 6 month follow-up period comparing children who were high or low antibody responders to each antigen or epitope.

4 Parasitemia detected by light microscopy.

### Association of Combined Epitope Responses with Protection from Clinical Malaria

To further understand the relevance of the different antibody responses in immunity, we examined associations with protection for combinations of responses. The risk of experiencing a clinical episode at any time within 6 months of sampling was investigated in Chonyi children with high responses to combinations of AMA1 responses compared to their low responder counterparts using Cox proportional hazards; analysis compared children who were high responders to a combination of two responses (AMA1/1F9, AMA1/2C5 or 1F9/2C5) versus those who were low responders to the same two responses. There was a greatly reduced risk of a clinical malaria episode during follow-up amongst children with high responses to AMA1 and 1F9 (HR = 0.15, P = 0.02) or high responses to AMA1 and 2C5 (HR = 0.21, P = 0.02) (Table 2); the hazard ratios were lower than those seen for single antibody responses. Those with combined high level responses to the 1F9 and 2C5 epitopes were also less likely to experience a clinical malaria episode, but this was of weak statistical significance (HR = 0.23, P = 0.07). When adjusted for age using multivariate analysis, the trends for protection remained for all combinations (HRs ranged from 0.29 to 0.36), but they were not statistically significant.

**Table 2:** Combined antibody responses to AMA1 and the risk of clinical malaria in the Chonyi cohort.

Antibody Reactivity Combinations^1^	Cox Proportional hazards		Adjusted Cox proportional hazards^2^	
	HR^3^	*P*-value	HR^3^	*P*-value
	(95% CI)		(95% CI)	
AMA1/1F9	0.15	0.02	0.29	0.16
	(0.03–0.71)		(0.05–1.61)	
AMA1/2C5	0.21	0.02	0.36	0.16
	(0.06–0.75)		(0.08–1.50)	
2C5/1F9	0.23	0.07	0.34	0.23
	(0.05–1.12)		(0.06–2.01)	

Notes.

1 Children were classified as high and low responders for antibodies based on being above or below the median level for the cohort. Analysis compared children who were high responders to both antigens versus those who were low responders to both.

2 Multivariable Cox proportional hazards adjusted for age.

3 Hazard ratios show the risk of *P. falciparum* malaria over a 6 month follow-up period.

### Association between Epitope-specific Antibodies and Inhibition of *P. falciparum* Growth

**Figure 3:**
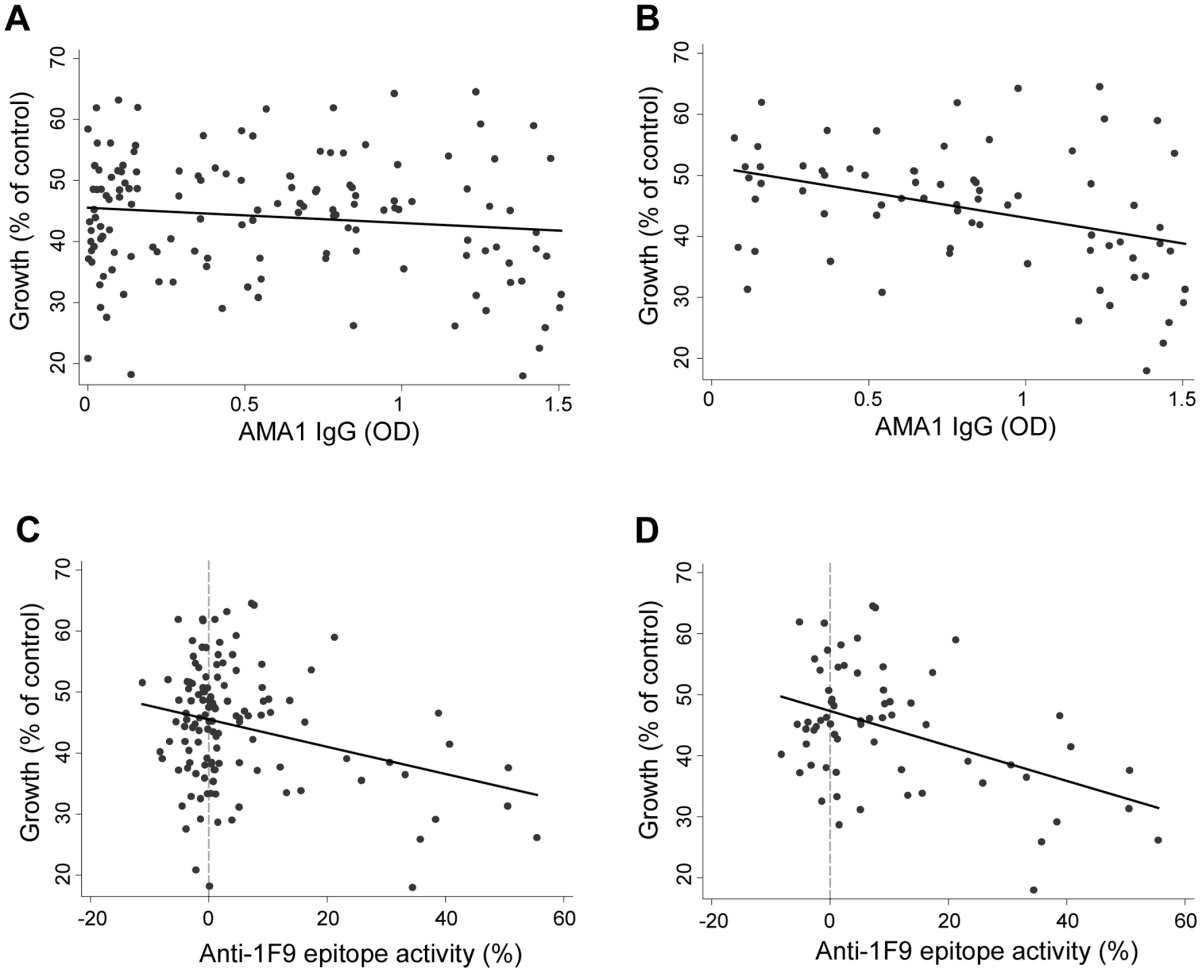
Association between antibody reactivity to AMA1 epitopes and *P. falciparum* growth in the Ngerenya cohort. (**A**) AMA1 antibody levels versus parasite growth for all samples (Spearman rank correlation, r = 0.09; P = 0.311), (**B**) AMA1 antibody levels and parasite growth for samples that were classified as high responders to schizont protein extract (antibody reactivity above the median), as a measure of higher exposure to blood-stage *P. falciparum* (r = −0.39; P = 0.001), (**C**) Anti-1F9 epitope activity (percentage inhibition of 1F9 mAb binding) and parasite growth in all samples (r = −0.11; P = 0.239). Positive 1F9 inhibition was defined as inhibition of 1F9 binding>(mean +2 standard deviations of negative controls = 6.19%). (**D**) Anti-1F9 epitope activity (percentage inhibition of 1F9 mAb binding) and parasite growth in samples with high levels of antibodies to AMA1 (defined as antibody levels above the median value of the group) (r = −0.25; P = 0.042). Parasite growth is expressed relative to controls (PBS). IgG binding to AMA1 is expressed as optical density (OD). Antibodies to the 1F9 epitope are expressed as a percentage of control (serum from malaria-naïve donors).

Since mAb 1F9 inhibits invasion [38], we hypothesised that acquired human antibodies recognizing the 1F9 epitope would inhibit parasite growth. Associations between levels of AMA1 IgG or anti-1F9 epitope activity and parasite growth in growth inhibition assays were investigated in 130 serum samples from children and adults in the Ngerenya cohort. Amongst all samples, there was no significant correlation between parasite growth and antibodies to AMA1 (r = −0.09, P = 0.311, Spearman’s rank correlation) (Figure 3A). As the magnitude of the response to AMA1 is strongly associated with prior exposure to *P. falciparum* (P<0.001, Kruskal Wallis), we then restricted our analysis to individuals with higher reactivity to schizont protein extract (defined as individuals with antibody reactivity above the median of the cohort); we reasoned that those with low levels of antibodies to AMA1 would have little growth inhibitory activity. Amongst individuals with high schizont reactivity, there was a significant correlation, with decreasing parasite growth as AMA1 IgG levels increased (r = −0.39, P = 0.001, Spearman’s rank correlation) (Figure 3B). High AMA1 antibody levels generally resulted in low parasite growth *in vitro*; however, there were some samples that showed high levels of AMA1 IgG and high levels of growth (Figure 3B). When the population was split into parasitemic and aparasitemic individuals, the negative correlation remained in the parasitemic individuals (r = −0.54, P = 0.001, Spearman’s rank correlation).

A negative correlation between parasite growth and serum antibody reactivity to the 1F9 epitope was expected, as the 1F9 monoclonal antibody inhibits parasite invasion. Although parasite growth in the presence of serum ranged from 10% –65% (relative to control), most individuals had low levels of antibodies to1F9 such that there was no significant correlation between anti-1F9 activity and parasite growth when including all samples (r = −0.11; P = 0.239, Spearman’s rank correlation) (Figure 3C). However, when our analysis was restricted to individuals with higher AMA1 antibody levels (defined as above the median) since the 1F9 response is a subset of the overall AMA1 response, there was a significant negative correlation between parasite growth and anti-1F9 activity (r = −0.25, P = 0.042, Spearman’s rank correlation) (Figure 3D), indicating that parasite growth declined as levels of antibodies to the 1F9 epitope increased. Samples with high reactivity to schizont protein extract also showed a significant negative correlation between anti-1F9 activity and growth (r = −0.33, P = 0.007, Spearman’s rank correlation) (results not shown). Those individuals with higher anti-1F9 activity consistently showed decreased parasite growth (Fig. 3D), in contrast to variable growth levels observed amongst individuals with high AMA1 antibodies. Sera from some Ngerenya individuals appeared to enhance (by up to 11%) the binding of the mAb 1F9 to its epitope (giving a relative inhibition value of less than zero; Figure 3). Six individuals showed enhanced 1F9 binding that was greater than 2 standard deviations of the non-exposed controls (samples with 1F9 competition of −6.2% or less). Of these samples, all were positive for IgG to AMA1 by ELISA and all samples showed some growth inhibitory activity (parasite growth ranged from 50.6 to 69.1%). The reason for apparently enhanced 1F9 binding to AMA1 in the presence of some samples is unknown. It is possible that the binding of serum antibodies to other sites on recombinant AMA1 might cause conformational modifications that make the 1F9 epitope more accessible to the 1F9 mAb thereby leading to enhanced 1F9 binding, but further studies are needed to better understand these effects and their significance. It has been shown that binding of the rhoptry neck protein RON2 to AMA1 is associated with structural changes in the region of AMA1 adjacent to the 1F9 binding site [52], and it is possible that antibodies may also induce such structural changes.

## Discussion

In this study, we have shown that individuals living in malaria-endemic settings acquire antibodies to polymorphic epitopes of AMA1, the inhibitory 1F9 epitope and the 2C5 epitope. Antibodies to both epitopes were acquired in association with increasing age and correlated with a marker of exposure to blood-stage *P. falciparum* and antibodies to full-length AMA1, and were generally higher in individuals with active parasitemia, although the pattern and strength of associations differed for the two epitopes. The demonstration that acquired human antibodies target the 1F9 epitope is particularly interesting, because 1F9 MAb blocks AMA1 function and inhibits invasion. Our findings suggest that individuals acquire antibodies that have a similar function to that of 1F9 mAb and target similar epitopes to inhibit invasion. Consistent with this, antibodies to the 1F9-epitope correlated with antibody growth-inhibitory activity, suggesting that people do acquire AMA1-specific growth-inhibitory antibodies through natural exposure. Presently it is not possible to quantify AMA1-specific inhibitory antibodies among individuals and examine the prevalence and pattern of acquisition of these antibodies. Therefore, these findings provide important insights into understanding the functional activity of antibodies to AMA1, which are believed to play a role in protective immunity [4], [17], [21]–[23]. In addition, recent studies suggest that the polymorphic C1-L region of AMA1, which includes the epitope of 1F9, is an important target of vaccine-induced protective human antibodies to AMA1 [27], [28]. Our findings that naturally-acquired antibodies target the 1F9 epitope further support the importance of the C1-L region in human immunity. Competition ELISAs using mAb 1F9 may be valuable in future studies of immunity to AMA1 and vaccine trials of AMA1.

High-level antibodies to AMA1, the 1F9 epitope and the 2C5 epitope were associated with significantly reduced risk of clinical malaria during follow-up in Chonyi children, and the association was strongest for antibodies to the inhibitory 1F9 epitope. However, antibodies were significantly and positively associated with increasing age, and associations with protection were no longer statistically significant after adjusting for age. Therefore, data on the associations between antibodies and protection should be interpreted with caution, and further studies are needed to investigate this association in other populations. These findings are broadly consistent with published studies on the same cohort, which showed antibody levels to the full length ectodomain of AMA1 (3D7 and FVO alleles) were associated with a reduced risk of malaria [17], [23]. When stratification by parasitemic status was performed in the current study, antibodies to AMA1 and the 2C5 epitope were only associated with protection in children with active infection, whereas in aparasitemic children there was no association. Further studies to understand this may be value. It is possible that protective antibodies to AMA1 and the 2C5 epitope are short-lived, but boosted when individuals are re-challenged by infection such that only parasitemic individuals have a sufficient level of antibodies to demonstrate an association with protection. Alternatively, it may be that active parasitemia has other immunologic effects, such as stimulation of cell-mediated immunity that is necessary for protective immunity in addition to antibodies. In contrast, 1F9 epitope antibodies were associated with a reduced risk of malaria regardless of infection status, which might indicate a more stable protective antibody response (although associations were not statistically significant for the smaller sample sizes that resulted from stratifying by parasitemic status). One of the previous studies conducted in the same cohort also found that associations between reduced risk of infection and antibodies against the AMA1 ectodomain were confined to parasitemic individuals [17]. Comparison of the current study with that of Polley et al [17], reveals that antibodies to AMA1 were more strongly associated with protection in the present study (RR = 0.35 compared to 0.71 in the univariate analysis). There are important differences in the study methodologies that might explain these findings. We compared antibody levels above and below the median whereas Polley et al compared antibody positive and antibody negative individuals as defined by levels of reactivity to AMA1 by sera from malaria non-exposed donors. Additionally, our study examined clinical protection in children and considered first episodes only, whereas Polley et al included adults and all episodes in their analysis.

Interactions between antibodies of differential specificities (either different malarial antigens or different epitopes of a single antigen) may be synergistic, antagonistic or neutral in their influence on disease outcome, and analysis of combinations of antibodies may reveal associations with protection that are not identified when responses to individual antigens are assessed [22], [23], [53]. In the present study, high level antibodies to the AMA1 ectodomain and the functional 1F9 epitope or AMA1 and the 2C5 epitope were associated with a strongly reduced risk of clinical malaria. While a combined high level response to 1F9 and 2C5 was also associated with a reduced risk, it had weak statistical significance. These data suggest that antibodies to other protective epitopes of AMA1 may work in concert with antibodies to the 1F9 and 2C5 epitopes, or that combined high responses to AMA1 and specific epitopes identifies a subset of responders with better protective immunity. It is interesting to speculate that combined high responses to AMA1 and the 1F9 epitope might identify those individuals with higher AMA1-inhibitory antibodies that contribute to parasitemia control and protection from clinical malaria.

Age is often considered as a potential confounding factor in studies assessing the association between immune responses and protection from malaria. In our study, we limited our survival analysis to children aged 1 to 10 years of age because prior studies have shown that this was the age range during which malaria immunity was acquired in this population [17], [41]. Adults were excluded to reduce the potential confounding effect of age because few adults experienced malaria during follow-up. When adjusting hazard ratios for the age of children, the trend for reduced risk of clinical malaria with higher antibody levels remained, but the associations were not statistically significant. The loss of statistical significance after adjusting for age is not unusual in these types of studies. Age is difficult to address as a confounding factor. It may be causally associated with the exposure variable (magnitude and quality of antibody responses are affected by age [54]), and may independently affect disease outcome through effects on other protective immune mechanisms and host factors implicated in disease pathogenesis. However, since acquisition of antibodies is dependent on cumulative exposure which is directly linked to age in endemic areas, adjustment for age may diminish any associations between protection and antibody levels. Results of adjusting for age in such studies should therefore be interpreted with caution.

The prevalence of 1F9 antibodies was low in both populations (less than 25%) despite the high prevalence of antibodies to the AMA1 ectodomain (above 75%), and was lower than prevalence of antibodies to the 2C5 epitope. The affinity of mAb 1F9 appears to be high (about one hundred times that of 2C5) [55], therefore only those sera with high affinity antibodies may inhibit binding of mAb 1F9 in competition ELISAs. The greater levels and prevalence of 2C5 reactivity observed here may in part reflect the lower affinity of the 2C5 mAb [55], allowing serum antibodies to more readily compete for binding. The positive correlation between serum inhibition of 2C5 mAb and 1F9 mAb binding indicates that antibodies to the two epitopes are generally co-acquired.

The lower prevalence of antibodies to 1F9 and 2C5 epitopes compared to the AMA1 ectodomain could reflect their polymorphic nature and the incidence of exposure due to 3D7-like alleles of AMA1. Although we do not have specific data on the prevalence of the 3D7 AMA1 allele at the study sites, 3D7 appears to be one of the more common AMA1 variants found in Africa (prevalence of 14% when AMA1 haplotypes are defined by amino acid changes within the C1-L region which includes key residues required for 1F9 binding) [36], [39]. Currently there is a limited understanding of antigenic diversity of AMA1, and how much antibody cross-reactivity there is between different AMA1 alleles. A recent study suggested that the antigenic diversity of AMA1 may be limited, and not as great as suggested by sequence analysis alone [33]. As the relationship between sequence polymorphism and antibody cross-reactivity is limited [33] and antibody cross-reactivity occurs between alleles [19], [33], it is difficult to relate the prevalence of specific AMA1 alleles in a population and the prevalence of antibodies. Information about the extent to which 1F9 and 2C5 antibodies cross-react with other strains is also limited, although they are known to recognise the D10 parasite line as well as 3D7 [38]. Low circulating levels of parasites expressing the 3D7 AMA1 allele in the study populations could account for the lower prevalence of anti-1F9 and anti-2C5 activity measured by competition ELISA but would not weaken the significance of the observed associations. Antibodies targeting the equivalent region in AMA1 variants that were more prevalent than 3D7 in these populations might show stronger associations with protection against clinical malaria; however, reagents to measure such antibodies are currently unavailable.

The ability of serum to inhibit 1F9 mAb binding correlated with growth inhibitory activity, although the association was only evident in sera with higher overall levels of antibodies to AMA1. This is the first study to demonstrate a link between naturally acquired human antibodies to the 1F9 epitope of AMA1, or any functional epitopes of AMA1, and growth inhibitory activity in a large set of human sera. Our findings support earlier studies which showed that the 1F9 mAb inhibits in vitro growth of *P. falciparum* and that naturally acquired human antibodies could compete with 1F9 mAb binding in competition ELISAs (in a small set of six plasma samples from Papua New Guinea adults) [38], [39]. Together with the association shown here between high levels of antibodies to the 1F9 epitope and clinical protection, these data suggest that the 1F9 epitope may be an important target for naturally acquired protective invasion inhibitory antibodies.

Further studies to confirm that antibodies to the 1F9 epitope are protective are needed and will have important implications for development of an AMA1 vaccine, since growth inhibitory antibodies targeting the 1F9 epitope are likely to be predominantly strain specific. The strain specific nature of vaccine-induced protective antibodies targeted to the region encompassing the 1F9 epitope was suggested by the recent clinical trial of the FMP2.1/AS02_A_ AMA1 vaccine [27], [28]. The association between antibodies to the 2C5 epitope and protection might be considered surprising, given that the 2C5 mAb has not been shown to inhibit parasite growth. However, affinity could be important for antibody function in the growth inhibition assay [55], so higher affinity naturally acquired antibodies to the 2C5 epitope might inhibit invasion and/or growth in vivo even though the 2C5 mAb does not. Alternatively natural antibodies to the 2C5 epitope may have some other protective mechanism of action, or 2C5 antibodies might be co-acquired with a protective response.

In conclusion, our studies suggest that naturally-acquired antibodies target the invasion inhibitory 1F9 epitope and non-inhibitory 2C5 epitope of AMA1, are acquired with increasing exposure to malaria, and show some association with a reduced risk of clinical malaria. The ability of acquired antibodies to inhibit the binding of mAb 1F9 and the correlation between these antibodies and growth inhibitory activity suggest that AMA1 is a target of naturally acquired growth-inhibitory antibodies in humans. Together, these findings provide further evidence supporting AMA1 as an important target of protective immunity. Competition ELISAs provide a useful tool for measuring the fine specificity of human malaria antibodies, and could be used in future studies of acquired immunity and AMA1 vaccine trials to assess invasion inhibitory antibodies targeting the 1F9 epitope. Additionally, these approaches may be valuable as markers of immunity for monitoring and surveillance of malaria in populations. Futures studies to further investigate these epitopes as targets of acquired and vaccine-induced immunity, particularly the inhibitory 1F9 epitope, define other functional and important epitopes, and better understand antigenic diversity of AMA1 will be valuable to further quantify the importance of AMA1 as an immune target and its priority as a vaccine candidate, and facilitate the development and testing of AMA1 vaccines.
